# Efficacy of Alum-Adjuvanted Peptide and Carbohydrate Conjugate Vaccine Candidates against Group A *Streptococcus* Pharyngeal Infection in a Non-Human Primate Model

**DOI:** 10.3390/vaccines12040382

**Published:** 2024-04-04

**Authors:** Tania Rivera-Hernandez, Diane G. Carnathan, Johanna Richter, Patrick Marchant, Amanda J. Cork, Gayathiri Elangovan, Anna Henningham, Jason N. Cole, Biswa Choudhury, Peter M. Moyle, Istvan Toth, Michael R. Batzloff, Michael F. Good, Paresh Agarwal, Neeraj Kapoor, Victor Nizet, Guido Silvestri, Mark J. Walker

**Affiliations:** 1 Consejo Nacional de Humanidades Ciencia y Tecnología, Unidad de Investigación Médica en Inmunoquímica, Hospital de Especialidades del Centro Médico Nacional Siglo XXI, Instituto Mexicano del Seguro Social, Mexico City 06720, Mexico; 2 School of Chemistry and Molecular Biosciences, The University of Queensland, St. Lucia, QLD 4072, Australia; 3 Emory Vaccine Center, Emory National Primate Research Center, Emory University, Atlanta, GA 30329, USA; diane.g.carnathan@emory.edu (D.G.C.);; 4 Institute for Molecular Bioscience, The University of Queensland, St. Lucia, QLD 4072, Australia; j.richter@uq.edu.au (J.R.); g.elangovan@imb.uq.edu.au (G.E.); 5 Vaxcyte Inc., San Carlos, CA 94070, USAparesh.agarwal@vaxcyte.com (P.A.); neeraj.kapoor@vaxcyte.com (N.K.); 6 Division of Ob/Gyn & Reproductive Sciences, Vc-Health Sciences-Schools, University of California San Diego, La Jolla, CA 92093, USA; anna.henningham@gmail.com (A.H.); bchoudhury@health.ucsd.edu (B.C.);; 7 School of Pharmacy, The University of Queensland, St. Lucia, QLD 4072, Australia; p.moyle@uq.edu.au; 8 Institute for Glycomics, Griffith University, Gold Coast, QLD 4222, Australia; m.batzloff@griffith.edu.au (M.R.B.);

**Keywords:** group A *Streptococcus*, *Streptococcus pyogenes*, J8 peptide, group A carbohydrate, vaccine

## Abstract

Vaccine development against group A *Streptococcus* (GAS) has gained traction in the last decade, fuelled by recognition of the significant worldwide burden of the disease. Several vaccine candidates are currently being evaluated in preclinical and early clinical studies. Here, we investigate two conjugate vaccine candidates that have shown promise in mouse models of infection. Two antigens, the J8 peptide from the conserved C-terminal end of the M protein, and the group A carbohydrate lacking *N*-acetylglucosamine side chain (ΔGAC) were each conjugated to arginine deiminase (ADI), an anchorless surface protein from GAS. Both conjugate vaccine candidates combined with alum adjuvant were tested in a non-human primate (NHP) model of pharyngeal infection. High antibody titres were detected against J8 and ADI antigens, while high background antibody titres in NHP sera hindered accurate quantification of ΔGAC-specific antibodies. The severity of pharyngitis and tonsillitis signs, as well as the level of GAS colonisation, showed no significant differences in NHPs immunised with either conjugate vaccine candidate compared to NHPs in the negative control group.

## 1. Introduction

Group A *Streptococcus* (GAS) is a human bacterial pathogen responsible for numerous clinical manifestations. Diseases caused by GAS vary widely in severity and outcome, ranging from mild infections such as pharyngitis, scarlet fever and impetigo to severe diseases such as sepsis, streptococcal toxic shock syndrome and necrotising fasciitis. Moreover, repeated mild infections can lead to autoimmune sequelae such as acute rheumatic fever (ARF) and rheumatic heart disease (RHD) [1,2]. Acknowledgment of the significant burden of disease caused by GAS infections has sparked renewed interest in the development of a vaccine that can safely prevent all the clinical manifestations caused by GAS [3,4,5,6].

Chemical conjugation of protective antigens, particularly carbohydrates, onto immunogenic carrier proteins has become a crucial tool in the prevention of bacterial infections. The use of conjugation as a vaccine platform has allowed the development of efficacious vaccines against several pathogens such as *Haemophilus influenzae*, *Streptococcus pneumoniae*, *Neisseria meningitidis* and *Salmonella enterica* serovar Typhi, with others in the pipeline [7,8]. A few vaccine conjugates candidates against GAS have also been developed and tested in preclinical studies [9,10,11]. We have previously reported protection in mice for two GAS conjugate vaccine candidates using the arginine deiminase (ADI) from this pathogen as a carrier protein [12], which itself has shown to provide protection in mouse infection models against GAS [13]. The first conjugate vaccine includes a peptide (J8) from the C-repeat region from the GAS M1 protein [14]. The J8 peptide is a component of other vaccine candidates, one of which has been tested in a phase I clinical trial [15]. The second conjugate vaccine contains the group A carbohydrate lacking *N*-acetylglucosamine side chain (ΔGAC), which is composed only of the backbone poly-rhamnose chain [16]. The removal of the *N*-acetylglucosamine side chain is intended to prevent the generation of potential cross-reactive antibodies recognising human heart tissue [17]. Previously, both J8 and ΔGAC were tested using diphtheria and tetanus toxoids as carrier proteins [9,15]. These proteins, even though proven to be immunogenic, are not GAS proteins, and therefore we decided to test a protein that would also contribute to specific immunity against GAS, such as ADI. In previous studies we showed that these conjugate vaccines (J8-ADI and ΔGAC-ADI), when formulated in alum, provided protection in a skin infection model in mice but did not provide protection using the humanised plasminogen mouse invasive disease model. Both conjugate vaccines triggered significant antibody responses in mice, as well as bactericidal antibodies using a whole blood killing assay [12]. Here, we report the immunogenicity and protective efficacy of these conjugate vaccines in a non-human primate (NHP) pharyngeal infection model. This NHP model has been employed for the evaluation of vaccine immunogenicity, contribution to protection against GAS colonisation and prevention of pharyngitis and tonsillitis signs [18]. Protection in the NHP model would build a strong case for advancement into clinical trials.

## 2. Materials and Methods

### 2.1. Bacterial Strains and Growth Conditions

Recombinant protein expression was carried out in *Escherichia coli* BL21 Star (DE3). *E. coli* was grown in Luria-Bertani medium (LB) with antibiotic selection. For NHP infection, *Streptococcus pyogenes* M1T1 strain 5448, an invasive clinical isolate [19], was grown in Todd–Hewitt medium supplemented with 1% (wt/vol) yeast extract (THY) and stored frozen (−80 °C) in aliquots of THY with glycerol (15%) until infection day.

### 2.2. Expression and Purification of ADI Protein

ADI (amino acids 1 to 411, D277A) previously cloned into the expression vector pET151/D-TOPO [13], was expressed in *E. coli* BL21 Star (DE3) cells and purified by immobilised metal ion affinity chromatography (IMAC) as previously reported [12]. Endotoxins were removed by washing the IMAC-bound protein with Triton X-114 [20]. Final protein concentration was determined using a Direct Detect infrared spectrometer (Millipore, Burlington, MA, USA) and endotoxin levels were measured using the Pierce Limulus amebocyte lysate (LAL) chromogenic endotoxin quantitation kit (Thermo Fisher Scientific, Waltham, MA, USA).

### 2.3. Peptide and Carbohydrate Conjugation to ADI

J8 peptide was commercially sourced (China Peptides Co., Hangzhou, China) and conjugated to purified ADI using *N*-(ε-maleimidocaproyloxy) sulfosuccinimide ester (Sulfo-EMCS; Thermo Fisher Scientific) following the manufacturer’s protocol as previously reported [12]. The ratio of J8 peptide to ADI carrier protein was determined using amino acid analysis (Australian Proteome Analysis Facility, Macquarie Park, NSW, Australia) and found to average three peptide molecules per ADI molecule as previously reported [12], which corresponds to 20 μg of J8 and 100 μg of ADI per dose. Streptococcal group A carbohydrate lacking the N-acetylglucosamine (GlcNAc) side chain (ΔGAC) was purified from the GAS 5448Δ*gacI* strain as previously reported [16] and conjugated to ADI by cyanylation as previously described [12]. Carbohydrate concentration in the conjugate was measured using the phenol–sulfuric acid method, and 15.3 μg of ΔGAC was found to be conjugated to 100 μg of ADI, which was the dose used to immunise NHPs. 

### 2.4. Animals

Ten rhesus macaques (8 female and 2 males) between 3.9 and 4.8 kg of body weight and between 3.2 and 3.5 years old were part of this study. Animals were held at the Emory National Primate Research Center and cared for following the National Institutes of Health guidelines [21]. Prior to commencing the study, NHPs were screened for current or recent GAS infection as previously reported [18]. NHPs were included in the study if, at the initial screening (day-7), they presented a throat swab negative for the presence of beta-haemolytic colonies, low levels of anti-DNAseB (<70 ng/mL) measured using an anti-DNase B antibody ELISA kit (American Research Products, Inc., Waltham, MA, USA), and the absence of anti-SLO antibodies as measured using an ASO latex agglutination assay (ASI). 

### 2.5. Immunisation and Challenge

NHP immunisation was carried out as described previously [18]. J8-ADI and ΔGAC-ADI conjugates were formulated in alum (Alhydrogel 2%; Brenntag Biosector, Frederikssund, Denmark) at a 1:1 ratio. M1 protein formulated in alum was used as a positive control whilst PBS formulated in alum was used as a negative control. Data from the positive and negative control groups has previously been reported [18]; nonetheless, the present study was conducted concurrently using the negative control group. Consequently, the data from both the M1 and PBS groups are included here for comparative purposes. Five NHPs per group were immunised with 100 μg of conjugate vaccine antigen (total protein) via intramuscular injection at week 0 (prime), week 8 (boost) and week 17 (boost). This immunization regime was chosen based on standard immunization regimes used in approved human vaccines for the pediatric population. Blood samples were collected prior to each immunisation, serum was obtained and used to monitor antigen-specific IgG responses. At week 20, NHPs were challenged with GAS M1T1 strain 5548 as previously described [18]. Briefly, anaesthetised macaques were infected by intranasal administration into the nares of 1 mL of the GAS suspension. Infected NHPs were monitored by veterinary staff on days 1, 2, 3, 7, 14, 21 and 28 post-infection. Body temperature, changes in body weight and white blood cell (WBC) counts were recorded at each time point. In addition, GAS colonisation of the pharynx was monitored by taking throat swabs at each of the aforementioned time points (Supplementary Table S1). Signs of pharyngitis and tonsillitis (Supplementary Table S2) were scored by veterinary staff at all follow-up time points using an established grading system [18,22]. At day 28 post-infection, NHPs were treated daily with penicillin G (30,000 U/kg) via intramuscular injection for 7 days. Anaesthesia using ketamine (10 mg/kg of body weight i.m.) or tiletamine-zolazepam (Telazol, Loveland, CO, USA) (5 mg/kg i.m.) was used for all blood and sample collections. 

### 2.6. ELISA

ADI- and J8 IgG-specific responses were measured as previously reported [12], endpoint titres were determined as the highest dilution of serum for which the absorbance was 3 times the standard deviations above the mean optical density of blank wells. For ELISA, biotinylated ΔGAC was produced in a two-step procedure. First, ΔGAC was activated with 1-cyano-4-dimethylaminopyridinium tetrafluoroborate (CDAP), followed by the addition of DBCO-PEG4-Amine to produce a DBCO-modified “activated” polysaccharide (APS) which was purified by tangential flow filtration on a regenerated cellulose membrane (Sartorius, Göttingen, Germany). The purified APS was treated with biotin azide (Click Chemistry Tools Cat# 1265-25) to generate biotinylated ΔGAC, which was purified using a spin desalting column (Thermo Scientific Zeba column, 7 kDa MWCO). The concentration of biotinylated ΔGAC was measured by anthrone assay [23]. ELISA to quantify serum reactivity against ΔGAC was undertaken using the method described above, using ELISA plates coated overnight with 10 µg/mL biotinylated ΔGAC (Vaxcyte Inc., San Carlos, CA, USA) diluted in Dulbecco’s phosphate-buffered saline at 4 °C. Absorbance values from naïve NHPs was subtracted from the absorbance values obtained for week 20 samples. This naïve-corrected data was used to determine the endpoint titres, as the highest dilution of serum for which the absorbance was 3 times the standard deviations above the mean optical density of blank wells.

### 2.7. Statistical Analysis

Differences in antigen-specific endpoint titres were analysed using the two-tailed Mann–Whitney U test with *p* of <0.05 considered statistically significant (GraphPad Prism 10). Cumulative scores for colonisation, pharyngitis, and tonsillitis were analysed using the Mantel–Cox log rank test, with a *p* value of <0.05 considered statistically significant (GraphPad Prism 10). 

### 2.8. Ethics Approvals

Animals were maintained in accordance with National Institutes of Health guidelines [21]. The experimental protocol was approved by the Emory University Institutional Animal Care and Use Committee.

## 3. Results

### 3.1. Antibody Responses to GAS Conjugate Vaccines

**Figure 1:**
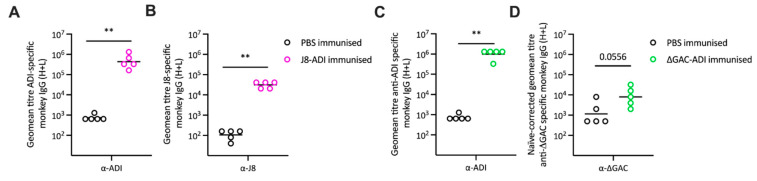
Antigen-specific IgG responses in NHP serum. Serum samples from J8-ADI-immunised (*n*  =  5), ΔGAC-ADI-immunised (*n* = 5) and PBS-immunised (*n* =  5) NHPs were collected prior to infection at week 20. In J8-ADI-immunised NHPs, IgG titres against ADI (**A**) and J8 (**B**) were significantly higher than in PBS-immunised NHPs (** *p*  <  0.01). Immunisation with ΔGAC-ADI induced a significantly increased IgG response against ADI (**C**), and an elevated, but not significant antibody response against ΔGAC ((**D**), *p* = 0.0556) compared to PBS-immunised NHPs. Individual antibody titres for each NHP are shown in circles. Lines represent geometric mean (geomean) titres.

Antigen specific responses were measured at week 20, prior to infection. Responses directed against ADI, the carrier protein, were significantly higher in samples from NHPs that received either of the conjugate vaccines, compared to NHPs in the PBS control group (Figure 1A,C). Similarly, the J8-specific IgG titres were significantly higher in the J8-ADI immunised NHPs compared to the PBS control group (Figure 1B). Specific antibody responses targeted against ΔGAC were difficult to detect, given that samples from naïve NHPs contained high levels of background binding (Supplementary Figure S1). To analyse ΔGAC-specific antibodies, titres from week 20 were calculated using matched naïve-corrected data for both the ΔGAC-ADI immunised group and the PBS immunised control group. The comparison showed that titres in the ΔGAC-ADI-immunised group were elevated compared to PBS-immunised NHPs (Figure 1D), although the difference fell short of reaching statistical significance (*p* = 0.0556).

### 3.2. Efficacy of GAS Conjugate Vaccines

**Figure 2:**
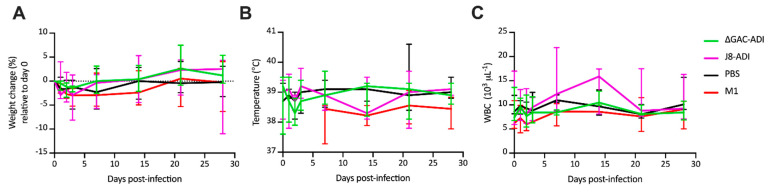
Animal welfare measures following infection of immunised NHPs. Welfare of J8-ADI (fuchsia lines) and ΔGAC-ADI (green lines) immunized NHPs during infection was monitored by veterinary staff by measuring weight change (**A**), rectal temperature (**B**) and white blood cell counts (**C**). Welfare measures for PBS (black lines) and M1 (red lines) immunised NHPs were previously reported [18] and are shown for comparative purposes. The temperature of M1-immunised NHPs was only measured from day 7 onwards. Lines represent the median values ± 95% CI.

**Figure 3:**
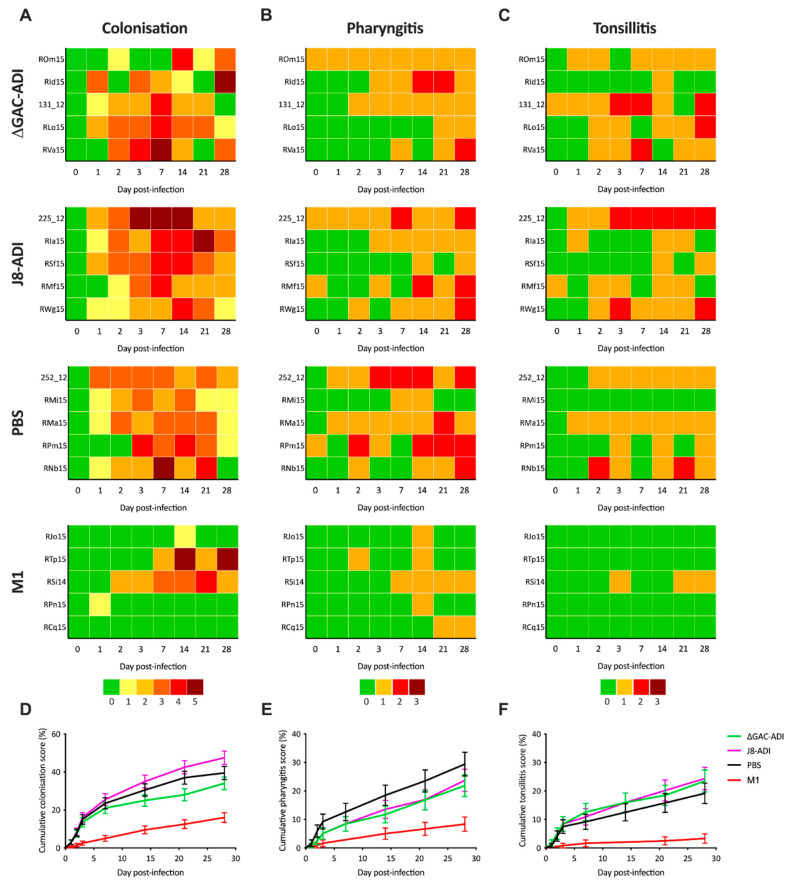
Immunisation with GAS conjugate vaccines does not reduce colonisation or signs in infected NHPs. Individual scores for colonisation (**A**), pharyngitis (**B**) and tonsillitis (**C**) following infection of immunised NHPs with 5 × 10^7^ CFU of GAS M1T1 5448. Individual NHP identifiers are indicated on the left. Log rank analysis of grouped cumulative scores for colonisation (**D**), pharyngitis (**E**) and tonsillitis (**F**) following GAS infection was performed to compare protection conferred by immunisation with J8-ADI (pink lines) and ΔGAC-ADI (green lines) to that of PBS-immunised control NHPs (black lines). Cumulative scores observed in a previously reported study [18] for PBS (black lines) and M1 (red lines) immunised NHPs are shown for comparative purposes. Values represent cumulative score percentages ± SE.

Immunised NHPs were infected at week 20 via intranasal administration of ~5 × 10^7^ colony-forming units (CFU) of GAS strain 5448 (range from 4.95 × 10^7^ to 5.22 × 10^7^ CFU). Changes in body weight, temperature and whole blood cell counts (WBC) were monitored over the course of infection (Figure 2A–C). For each parameter there was no significant difference between the conjugate vaccine groups compared to the PBS control group. Colonisation and signs were also assessed at days 0, 1, 2, 3, 7, 14 and 28 (Figure 3A–C). NHPs from the three groups were colonised by GAS for most of the duration of the experiment. There was no significant difference in colonisation levels of NHPs immunised with ΔGAC-ADI and J8-ADI compared to the PBS-alum negative control (Figure 3D). Analysis of cumulative pharyngitis scores (Figure 3E) showed that scores for ΔGAC-ADI and J8-ADI were lower than scores for the PBS-immunised group, however this difference was not statistically significant (*p* = 0.1645 for ΔGAC-ADI; *p* = 0.2853 for J8-ADI). Finally, the cumulative score analysis showed no significant difference in the severity of tonsillitis signs between the groups that received the conjugate vaccine and the control group (Figure 3F).

## 4. Discussion

There is a well-recognised need for a vaccine to address infections caused by GAS. A range of vaccine candidates are in the development pipeline, currently being tested in preclinical or early clinical stages [7,24,25,26,27,28]. Preclinical evaluation of GAS vaccines has represented a major challenge, given that the most common clinical manifestation, pharyngitis, cannot be modelled in mice. In addition, pharyngitis is the most predominant clinical manifestation caused by GAS, with a global estimate of 288.6 million cases every year in children between the ages of 5 and 14 [29]. For this reason, expert working groups and the World Health Organization proposed pharyngitis as a potential vaccine efficacy trial endpoint in the preferred product characteristics for GAS vaccines [30,31]. There are reports of non-experimental GAS colonisation of rhesus macaques [32], making this species a good candidate for a GAS upper respiratory tract infection model. This model offers the possibility of evaluating vaccine protection using GAS colonisation and signs of pharyngitis and tonsillitis as protective endpoints, which closely resemble signs in humans. The infecting dose used in the NHP model, 5 × 10^7^ CFU administered intranasally in a 1 mL suspension, was determined to be optimal in a pilot study previously reported [18]. This dose is higher than the one reported to cause pharyngitis in the GAS human challenge model that resulted in an 85% success rate [33]; however, we have previously reported that vaccine-mediated immunity can be observed using M1 protein as a vaccine antigen with this infecting dose in the NHP model. The two conjugate vaccine candidates (ΔGAC-ADI and J8-ADI) tested in this study had shown conflicting results regarding protective efficacy in two different mouse infection models [12]. We therefore tested each conjugate vaccine in the NHP model to form a more accurate perspective of the protective efficacy of these candidates.

Both vaccines were immunogenic, as assessed by ELISA antibody responses, particularly for the protein (ADI) and peptide (J8) components of the vaccine candidates. In the case of the ΔGAC-ADI carbohydrate, we found high levels of background ΔGAC-specific antibodies, even in naïve NHP samples. This is in accordance with data that show that naïve serum samples from cynomolgus macaques have high antibody binding to rhamnose [34], the subunit component of ΔGAC. Cynomolgus macaques are closely related to the rhesus macaques used in this study, hence this result is not surprising. It is important to highlight that, to a lesser extent, we also observed this high background binding from naïve samples in mouse samples previously [12]. Despite high background binding, we could observe elevated levels of ΔGAC antibodies in samples from ΔGAC-ADI-immunised NHPs compared to PBS controls when absorbance values from baseline samples were subtracted for each animal; however, this increase did not reach statistical significance. Unfortunately, we were not able assess other key factors that participate in the immune response against GAS. In future work, it will be crucial to measure the bactericidal activity of the serum samples, the presence of other immunoglobulin isotypes such as IgA, activation of cellular immunity and the generation of immune memory.

Following intranasal infection with GAS M1T1, none of the conjugate vaccines offered significant levels of protection in terms of the three physiological parameters that were monitored, colonisation and signs of pharyngitis and tonsillitis. The absence of significant differences in the severity of pharyngitis and tonsillitis signs, as well as the level of GAS colonisation in NHPs immunised with either conjugate vaccine candidate compared to the PBS-alum negative control, does not imply that further development of these candidates should be abandoned. Instead, it suggests the need for optimization in formulation, including strategies to enhance antigen density through optimised conjugation, exploration of alternative carrier proteins and consideration of novel adjuvants to effectively potentiate and shape the immune response. For example, a novel click-chemistry-based technology [35] allows precise bioconjugation of a carbohydrate antigen such as ΔGAC to preserve key functional epitopes of the carrier protein, which might otherwise be altered or shielded by the indiscriminate targeting of lysine sidechain ε-amine sites during reductive amination. We have also demonstrated that the use of experimental adjuvants that stimulate a more balanced Th1/Th2 immune response can significantly improve protective efficacy in a mouse model of invasive disease [36]. We consider that the use of such optimised conjugates and/or adjuvants should be assessed in the NHP model described here. Lastly, the dose of delivered antigen was based on the amount of total protein (100 μg) for both vaccine conjugates, without prior dose escalation studies or optimisation of the immunisation schedule. These are aspects that could certainly modify the immune response to the vaccine antigens, however they remain difficult to study in NHPs due to limited access to specimens.

## 5. Conclusions

This study highlights the complexities and challenges inherent in the development of an effective vaccine against GAS. While both J8-ADI and ΔGAC-ADI vaccine candidates elicited immunogenic responses in the NHP model, they did not elicit significant protection against GAS infection in terms of reduced colonisation or alleviation of pharyngitis and tonsillitis signs. These findings underscore the need for continued refinement in vaccine design, including potential improvements in antigen density, alternative carrier proteins, more effective adjuvants or expanding the valency with additional immunogenic GAS antigens. Despite the lack of significant protective efficacy observed in this study, the insights gained may help guide future research directions in the ongoing global effort to develop a safe and efficacious GAS vaccine.

## Data Availability

Data supporting reported results is available from the authors upon request.

## Supplementary Materials

The following supporting information can be downloaded at: https://www.mdpi.com/article/10.3390/vaccines12040382/s1, Figure S1: Anti-ΔGAC and anti-ADI antibodies in naïve NHP serum samples. Levels of anti-ΔGAC IgG antibodies in serum samples from naïve NHPs were from 1 to 2-log higher than baseline antibodies for protein antigens, such as ADI; Table S1: Colonisation scoring system [37]. Table S2: Pharyngitis and tonsillitis signs scoring system [22].
