# Supplementary material for: Efficacy of Alum-Adjuvanted Peptide and Carbohydrate Conjugate Vaccine Candidates against Group A Streptococcus Pharyngeal Infection in a Non-Human Primate Model
Source: Vaccines (Basel). 2024 Apr 4;12(4):382. doi: 10.3390/vaccines12040382 (PMC11054769; doi:10.3390/vaccines12040382)
Supplement: Supplementary file 1 [file vaccines-12-00382-s001.zip › vaccines-2897171-supplementary.pdf]

Supplementary information

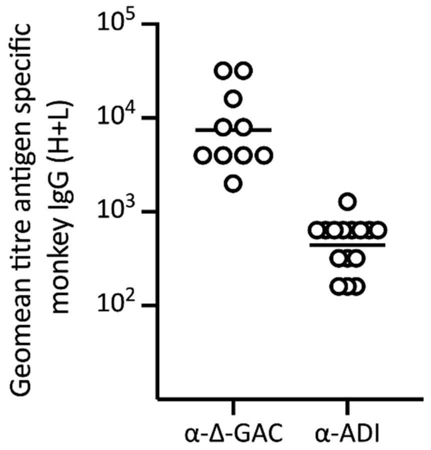

Supplementary figure S1. Anti-ΔGAC and anti-ADI antibodies in naïve NHP serum samples. Levels of anti-ΔGAC IgG antibodies in serum samples from naïve NHPs were from 1 to 2-log higher than baseline antibodies for protein antigens, such as ADI.

Supplementary table S1. Colonisation scoring system (Dunne, 2013).

Colonisation	Score
No β-haemolytic colonies	0
<10 β-haemolytic colonies in 1st plate quadrant	1
>10 β-haemolytic colonies in 1st plate quadrant	2
>10 β-haemolytic colonies in 1st and 2nd plate quadrants	3
>10 β-haemolytic colonies in 1st, 2nd and 3rd plate quadrants	4
>10 β-haemolytic colonies in 1st, 2nd, 3rd and 4th plate quadrants	5

Dunne, E.M., J.L. Marshall, C.A. Baker, J. Manning, G. Gonis, M.H. Danchin, P.R. Smeesters, C. Satzke & A.C. Steer, (2013) Detection of group a streptococcal pharyngitis by quantitative PCR. BMC Infectious Diseases 13: 312.

Supplementary table S2. Pharyngitis and tonsillitis signs scoring system (Skinner, 2011).

Pharyngitis	Tonsillitis	Score
Normal	Normal tonsillar and oropharyngeal space	0
Mild erythema with hyperemic blood vessels	Aproximate 25% increase in tonsil size; 0-25% occlusion of oropharyngeal space	1
More intense erythema and palatal petechiae	Approximate 50% increase in tonsil size; 25-50% occlusion of oropharyngeal space	2
Intense erythema with palatal petechiae and exudative tonsillitis	Approximate 75% increase in tonsil size; 50-75% occlusion of oropharyngeal space	3

Skinner, J.M., I.C. Caro-Aguilar, A.M. Payne, L. Indrawati, J. Fontenot & J.H. Heinrichs, (2011) Comparison of rhesus and cynomolgus macaques in a *Streptococcus pyogenes* infection model for vaccine evaluation. Microbial pathogenesis 50: 39-47.
